# miR-223-3p Inhibits Antigen Endocytosis and Presentation and Promotes the Tolerogenic Potential of Dendritic Cells through Targeting Mannose Receptor Signaling and Rhob

**DOI:** 10.1155/2020/1379458

**Published:** 2020-06-18

**Authors:** Hao-Cheng Tang, Yin-Yan Lai, Jing Zheng, Hong-Yan Jiang, Geng Xu

**Affiliations:** ^1^Otolaryngology-Head & Neck Surgery, Nanfang Hospital of Southern Medical University, 1838 Guangzhou North Avenue, Guangzhou 510515, China; ^2^Otorhinolaryngology Hospital, The First Affiliated Hospital of Sun Yat-sen University, 58 Zhongshan Road II, Guangzhou 510080, China; ^3^Hospital of Otorhinolaryngology Head and Neck Surgery, Hainan General Hospital, 19 Xiuhua Road, Haikou 570311, China; ^4^Department of Otorhinolaryngology Head and Neck Surgery, Xiamen Humanity Hospital, 3777 Xianyue Road, Xiamen 36100, China

## Abstract

**Background:**

The role of miR-223-3p in dendritic cells (DCs) is unknown. This study is aimed at investigating the effect of miR-223-3p on the antigen uptake and presentation capacities of DCs and the underlying molecular mechanism.

**Methods:**

FITC-OVA antigen uptake and cell surface markers in bone marrow-derived DCs (BMDCs) were analyzed by flow cytometry. BMDCs were transfected with the miR-223-3p mimic or inhibitor. Cytokine levels were determined by ELISA. CD4+ T cell differentiation was determined by mixed lymphocyte culture assay.

**Results:**

OVA treatment significantly downregulated miR-223-3p in BMDCs. The miR-223-3p mimic significantly inhibited OVA-induced antigen uptake and surface expression of MHC-II on BMDCs (*P* < 0.01). The miR-223-3p mimic increased TGF-*β*1 production in OVA-treated DCs (*P* < 0.01). Mixed lymphocyte reaction showed that the miR-223-3p mimic significantly promoted Treg cell differentiation. In addition, the miR-223-3p mimic significantly upregulated CD103 in DCs, indicating the promotion of tolerogenic DCs. The miR-223-3p mimic downregulated Rhob protein in OVA-induced DCs. Rhob knockdown significantly suppressed the ability of FITC-OVA endocytosis (*P* < 0.01) and surface MHC-II molecule expression (*P* < 0.01) in BMDCs, promoting promoted Treg cell differentiation. Mannose receptor (MR) knockdown significantly upregulated miR-223-3p, downregulated Rhob protein in OVA-treated DCs, inhibited the FITC-OVA endocytosis and surface MHC-II expression in BMDCs, and promoted Treg cell differentiation (all *P* < 0.01).

**Conclusion:**

These data suggest that miR-223-3p has an inhibitory effect on the antigen uptake and presentation capacities of BMDCs and promotes Treg cell differentiation, which is, at least partially, through targeting MR signaling and Rhob.

## 1. Introduction

Dendritic cells (DCs) are the most potent antigen-presenting cells for inducing primary adaptive responses and maintaining self-tolerance [1]. DCs can uptake foreign antigens which were degraded into smaller peptides and subsequently loaded onto major histocompatibility complex (MHC) on the surface, presenting peptide fragments for recognition by antigen-specific T cells [2, 3]. Class I MHC (MHC-I) is recognized by cytotoxic CD8+ T cells, while Class II MHC (MHC-II) is recognized by helper CD4+ T cells [4]. During antigen-specific T cell activation, DCs can produce cytokines, such as IL-1*β*, to facilitate the antigen-specific T cell proliferation and differentiation into one of the T cell phenotypes [5]. In addition to CD4 helper and CD8 cytotoxic T cells, DCs are capable of inducing regulatory T (Treg) cell differentiation, which plays a role in controlling the immune tolerance and homeostasis [6, 7].

miRNAs are a class of small (20–25 nucleotides in length), single-stranded, non-coding, RNA molecules that exert function via binding mRNA targets, leading to their degradation or translational suppression [8]. miRNAs have been shown to be involved in a variety of important biological functions, such as cell development, apoptosis, signal transduction, and pathogenic conditions [8, 9]. It has been predicted that up to 60% of human genes may be controlled by miRNAs [10]. Increasing evidence shows that miRNAs can be important regulators of immune responses, such as miR-146a [11], miR-29 [12], and miR-155 [13]. miRNAs have also been shown to be involved in DC development and DC function regulation, including the production of cytokines, differentiation, and homeostasis via affecting specific targets [14]. For instance, Naqvi et al. have demonstrated that overexpression of miR-24, miR-30b, and miR-142-3p attenuates uptake and processing of soluble antigen ovalbumin (OVA) in DCs [15]. Our preliminary high-throughput sequencing results showed that OVA treatment results in altered expression of miR-223-3p in DCs, suggesting miR-223-3p may take part in regulating DC function.

miR-223-3p is involved in innate immune responses by regulating myeloid differentiation and granulocyte functions [16, 17]. miR-223-3p has been shown to be involved in regulating the differentiation and function of DCs [18–20]. Although the function of miR-223-3p in adaptive immune responses has been demonstrated, its role in antigen uptake and presentation is unknown. Therefore, the purpose of this study was to investigate the effect of miR-223-3p on the antigen uptake and presentation capacities of BMDCs and the underlying molecular mechanism.

## 2. Materials and Methods

### 2.1. Culture and Transfection of Mouse DCs

BALB/C mice (6-8 weeks) were purchased from Guangdong Medical Experimental Animal Center (Guangzhou, China) and bred in specific pathogen-free conditions. Bone marrow-derived DCs (BMDCs) were isolated from BALB/C mice as previously described [21] with some modifications. Briefly, bone marrow progenitors were cultured in RPMI 1640 (HyClone, USA) supplemented with 10% FBS (Gibco, USA), 10 ng/ml GM-CSF (PeproTech, London, UK), and 10 ng/ml IL-4 (PeproTech, London, UK). Immature BMDCs were harvested at 5-6 days after differentiation. BMDC medium contains glutamine, but no antibiotics. DCs were positively selected using CD11c magnetic microbeads (Miltenyi Biotec, Bergisch Gladbach, Germany). All animal experiments were performed in accordance with the National Institutes of Health Guide for the Care and Use of Laboratory Animals and were approved by the Scientific Investigation Board of Sun Yat-Sen University (Guangzhou, China).

### 2.2. Quantitative RT-PCR Detection

Total RNA was extracted from DCs using TRIzol reagent (Invitrogen) according to the manufacturer's protocol and was reversely transcripted to cDNA with specific stem-loop primers by using a reverse transcription kit (Tiangen Biotech Co., Ltd., China). Quantitative PCR analysis of miRNAs was performed using an SYBR qPCR kit (Toyobo, Japan) and the ABI Prism 7500 Sequence Detection System (Applied Biosystems, Carlsbad, CA, USA). The relative expression level of miRNAs was normalized to the internal control (U6) using the 2^-*ΔΔ*Ct^ cycle threshold method. The primers for miR-223-3p and U6 were purchased from Guangzhou RiboBio Co., Ltd. (Guangzhou, China).

### 2.3. miRNA Mimic and Inhibitor

The sequences of the miR-223-3p mimic/inhibitor and their controls are shown in Table 1. All of the oligonucleotides were synthesized and purified with high-performance liquid chromatography by Shanghai GenePharma (Shanghai, China). For miRNA mimic and inhibitor transfection, 2 × 10^6^ cells/well were seeded onto a 6-well plate. Cells were transfected with the mimic and inhibitor or the negative control of the mimic and inhibitor using Lipofectamine 2000 (Invitrogen, USA) according to the manufacturer's protocol. The final concentration of Lipofectamine 2000 was 10 *μ*l/250 ml.

### 2.4. RNA Interference

For RNAi transfection, 2 × 10^6^ cells/well were seeded onto a 6-well plate. siRNA duplexes were transfected into BMDCs at a final concentration of 10 nM using Lipofectamine 2000. The sequences for the siRNA-Rhob, siRNA-mannose receptors (MRs), and negative control are summarized in Table 2.

### 2.5. Flow Cytometry

For cell surface and intracellular marker analysis, 10^6^ cells were incubated with fluorescent-conjugated antibodies in labeling solution (eBioscience, USA). The fluorescent-conjugated antibodies used in this study included PE-conjugated anti-mouse CD11c, FITC-conjugated anti-mouse CD11c, FITC-conjugated anti-mouse CD86, FITC-conjugated anti-mouse MHC-II, APC-conjugated anti-mouse CD80, PE-CY5-conjugated anti-mouse CD40, APC-conjugated anti-mouse CD4, FITC-conjugated anti-mouse IL-17A, FITC-conjugated anti-mouse Foxp3, PE-conjugated anti-mouse GATA-3, PE-conjugated anti-mouse T-bet antibodies, and FITC-conjugated anti-mouse CD103 (integrin alpha E) (all purchased from eBioscience, USA). Fluorescent-conjugated, isotype-matched, irrelevant antibodies were used to establish background fluorescein levels. Flow cytometry analysis was conducted on a FACSCalibur (BD Biosciences, USA), and FACSCalibur software (BD Biosciences) was used to analyze the flow data. DCs were gated for PE-CD11c, and then FITC-MHC-II expression and endocytic FITC-OVA levels were analyzed. MLR-lymphocytes were gated for APC-CD4, and then FITC-Foxp3 was analyzed.

### 2.6. Endocytosis Assay

The suspended and semiadherent cells were collected and washed 3 times with precooled PBS, and 1.5 × 10^6^ cells were resuspended in FITC-OVA (100 *μ*g/ml, diluted in the medium). Since the cells were washed by precool PBS, to eliminate the effect of low temperature (4°C) on DC endocytosis, the cells were incubated at 37°C or 4°C (control) for 30 min. The cells were washed three times with precooled PBS, followed by incubating with the PE-labeled CD11c antibody and the corresponding isotype control antibody (1 *μ*g/ml) at 4°C for 30 min. After washing twice with 500 *μ*l PBS, the cells were subjected to flow cytometry analysis. The endocytic activity was calculated by the following equation: Δ mean fluorescence intensity  (MFI) = MFI  of  FITC‐OVA uptake 37°C − MFI of FITC‐OVA uptake 4°C.

### 2.7. Cytokine Measurement

The cytokine levels of TGF-*β*1, IL-6, IL-10, and IL-12 in the supernatants were determined by ELISA kits (R&D, USA) according to the manufacturer's protocols.

### 2.8. Mixed Lymphocyte Culture (MLC) for CD4+ T Cell Differentiation

Splenic CD4+ T cells from spleen mononuclear cells of BALB/C mice were negatively selected via magnetic cell separation (Miltenyi) and were used as the responders. DCs were transfected with the miR-223-3p mimic and inhibitor or their controls and then washed with PBS and resuspended in a new round-bottom 96-well plate at a density of 10^4^ cells/well. T cells (10^5^ cells/well) were added to each to coculture with DCs in the presence of OVA (100 *μ*g/ml, Sigma-Aldrich, St. Louis, MO, USA) at a ratio of 1 : 10 (DC/T cells) for 4 days. After which, cells were stained with APC-conjugated anti-mouse CD4 and incubated with fixation/permeabilization buffer (eBioscience), followed by intracellular staining with FITC-conjugated anti-Foxp3, anti-T-bet+, anti-GATA-3, or anti-IL-17A antibodies and flow cytometry analysis.

### 2.9. Western Blot

DCs were lysed in cell lysis buffer (Millipore, USA) with protease inhibitor cocktail (Sigma, USA). Protein concentration was determined by the Bradford method. Equal amounts of proteins were separated by 9% SDS-PAGE and then transferred to nitrocellulose membranes (Millipore). Primary antibodies Rhob (anti-mouse, Cell Signaling, USA), Rhoa (anti-rabbit, Cell Signaling), Rac-1 (anti-rabbit, Cell Signaling), CDC42 (anti-mouse, Santa Cruz, USA), MR (anti-goat, Abcam, UK), and GAPDH (anti-mouse, Santa Cruz, USA) and secondary antibodies HRP-labeled goat anti-mouse IgG (Jackson ImmunoResearch, USA), HRP-labeled goat anti-rabbit IgG (Jackson ImmunoResearch, USA), and HRP-labeled rabbit anti-IgG (Jackson ImmunoResearch) were all used in the Western blot. Protein detection was performed using a chemiluminescence system (SuperSignal West Pico; Pierce, Rockford, IL, USA).

### 2.10. Statistical Analysis

For all experiments, the data was presented as mean ± SD of three independent experiments (*n* = 3 for each group) and was compared between two groups using a paired *t*-test. For comparison among more than two groups, one-way ANOVA with post hoc Tukey's HSD test was performed for multiple comparisons. All analyses were performed with SPSS v16.0 (SPSS, Chicago, IL, USA). A *P* < 0.05 was considered statistically significant.

## 3. Results

### 3.1. OVA Treatment Downregulated miR-223-3p in BMDCs

**Figure 1:**
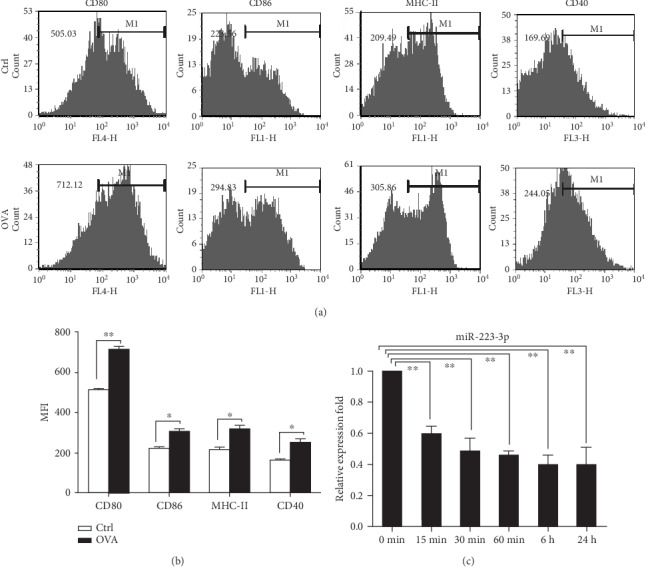
miR-223-3p was downregulated in OVA-induced DCs. (a) Mouse immature DCs were treated with 100 *μ*g/ml OVA for 24 h and then stained with specific Abs against MHC-II, CD80, and CD86 for flow cytometry analysis. (b) The bar chart indicated the MFI of the DCs in each group. *t*-test, ^∗^*P* < 0.05, compared to the control group. (c) miR-223-3p expression in OVA-treated immature DCs. Immature DCs were incubated with OVA (100 *μ*g/ml) for 24 h. Cells were collected at the indicated time points, and miR-223-3p expression was determined by RT-PCR. Data were shown as mean ± SD of three independent experiments. Post hoc Tukey's HSD test, ^∗^*P* < 0.05, ^∗∗^*P* < 0.01, compared to the 0 min control.

Immature DCs from mouse bone marrow can be induced to mature *in vitro* via OVA stimulation, which was characterized by elevated surface expression of MHC-II, CD80, CD86, and CD40 (Figures 1(a) and 1(b)). Our preliminary high-throughput sequencing results showed that OVA treatment induces a decrease in the miR-223-3p level in DCs (unpublished data); therefore, the miR-223-3p expression kinetics in OVA-treated DCs was determined by qPCR analysis. As shown in Figure 1(c), OVA treatment significantly downregulated miR-223-3p in DCs from 0 min to 24 h in a time-dependent manner (*P* < 0.01).

### 3.2. miR-223-3p Suppressed OVA Endocytosis and OVA-Mediated Surface Expression of MHC-II Molecules on BMDCs

**Figure 2:**
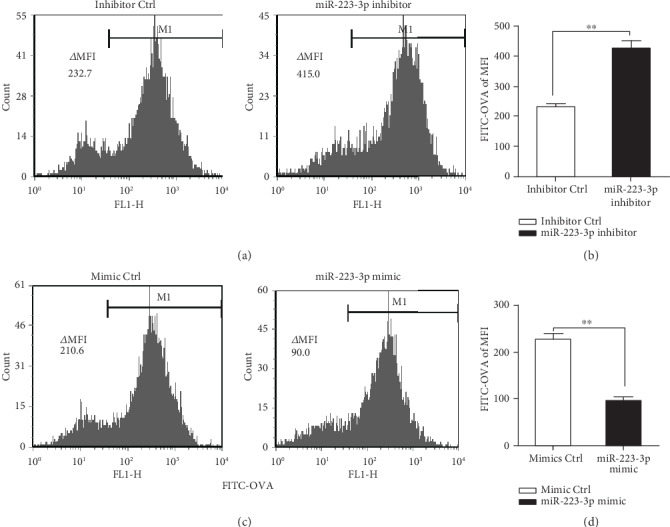
miR-223-3p suppressed OVA endocytosis of BMDCs. Mouse immature DCs were transfected with the miR-223-3p inhibitor (a, b), miR-223-3p mimic (c, d), and the corresponding controls at a final concentration of 50 nM. After 24 h of transfection, DCs were incubated with FITC-OVA for 30 min and the endocytic activity (FITC-OVA uptake) of BMDCs was measured by flow cytometry. (b, d) The bar chart indicated the MFI in the gate of CD11c+ cells in each group. Similar results were obtained in three independent experiments. Data were shown as mean ± SD. *t*-test, ^∗^*P* < 0.05, ^∗∗^*P* < 0.01.

The changes in the miR-223-3p level led us to consider whether it participates in regulating the biological function of DCs. To investigate the role of miR-223-3p in DCs, the miR-223-3p mimic and miR-223-3p inhibitor were adopted. RT-PCR showed that miR-223-3p mimic transfection significantly elevated miR-223-3p expression in DCs, whereas miR-223-3p inhibitor transfection significantly reduced miR-223-3p expression as compared with the control group (Supplementary Fig. S1A), suggesting a high efficiency of the miR-223-3p mimic and inhibitor. To determine if miR-223-3p regulates the antigen endocytosis capacity of BMDCs, the cells were transfected with the miR-223-3p inhibitor/mimic before FITC-OVA incubation. As shown in Figures 2(a) and 2(b), miR-223-3p inhibition significantly enhanced endocytic uptake of FITC-OVA in BMDCs as compared with the inhibitor control group (*P* < 0.01). By contrast, the miR-223-3p mimic markedly inhibited the endocytic activity of BMDCs (*P* < 0.01, Figures 2(c) and 2(d)).

**Figure 3:**
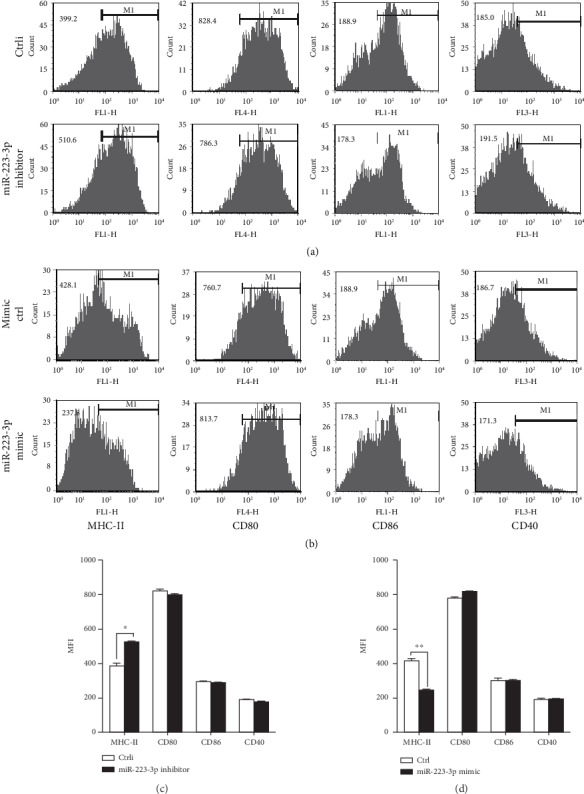
miR-223-3p suppressed surface molecule MHC-II expression on the OVA-induced BMDCs. Mouse immature DCs were transfected with the miR-223-3p inhibitor (a), inhibitor control, miR-223-3p mimic (b), or mimic control at a final concentration of 50 nM. After 24 h, DCs were stimulated with 100 *μ*g/ml OVA for 24 h and then stained with specific Abs against MHC-II, CD80, CD86, and CD40 and then analyzed by flow cytometry. The bar chart indicated the MFI in the gate of CD11c+ cells in each group. Similar results were obtained in three independent experiments. Data were shown as mean ± SD. *t*-test, ^∗^*P* < 0.05, ^∗∗^*P* < 0.01.

Next, we examined the effect of miR-223-3p on the expression of surface antigen presentation molecule MHC-II. Flow analysis showed that the miR-223-3p inhibitor significantly upregulated MHC-II (*P* < 0.01, Figure 3(a)) but did not affect CD80, CD86, and CD40 (Figure 3(a)). By contrast, the miR-223-3p mimic significantly downregulated surface antigen presentation molecule (MHC-II) in BMDCs (*P* < 0.05, Figure 3(b)). These results demonstrated that miR-223-3p inhibited OVA endocytosis and OVA-mediated surface expression of MHC-II in BMDCs.

### 3.3. miR-223-3p Increased TGF-*β*1 Production in OVA-Treated DCs and Promoted Treg Cell Differentiation

**Figure 4:**
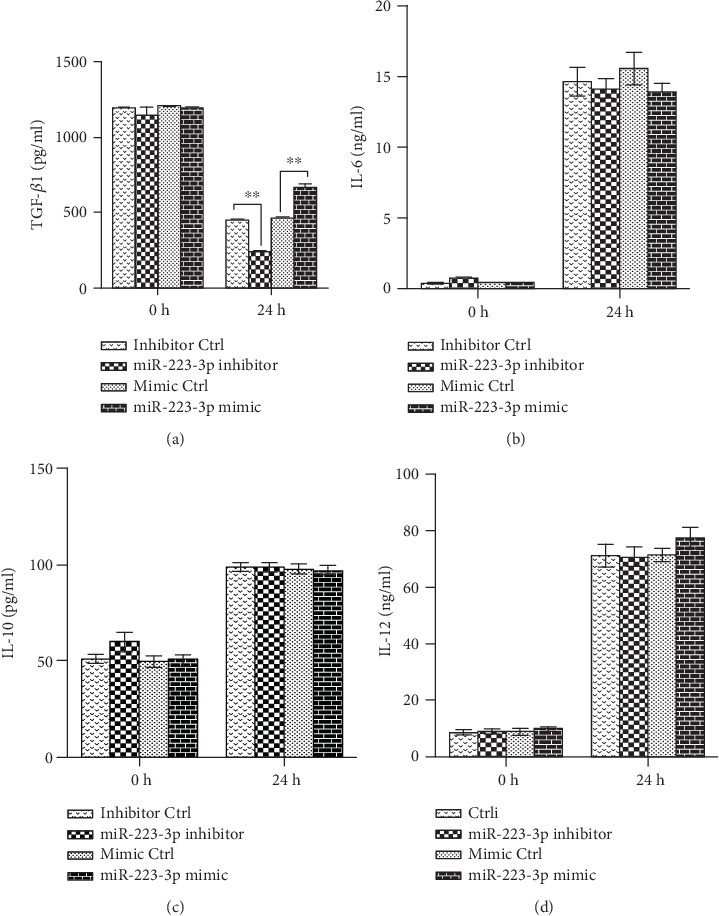
miR-223-3p regulated TGF-*β*1 production in OVA-induced DCs. Immature mouse DCs transfected with the miR-223-3p inhibitor, inhibitor control, miR-223-3p mimic, or mimic control at a final concentration of 50 nM. After 24 h, DCs were stimulated with 100 *μ*g/ml OVA for 24 h. TGF-*β*1 (a), IL-6 (b), IL-10 (c), and IL-12 (d) levels in the supernatants were measured by ELISA. Data were shown as mean ± SD of three independent experiments. Post hoc Tukey's HSD test, ^∗^*P* < 0.05, ^∗∗^*P* < 0.01.

The production of inflammatory cytokines and the upregulation of cell surface molecules, including MHC and costimulatory molecules in DCs, are essential for the priming of naive T cells [22]. To investigate if miR-223-3p plays a role in OVA-induced cytokine production in DC and T cell differentiation, miR-223-3p mimic or inhibitor transfections were performed. As shown in Figure 4(a), the miR-223-3p mimic remarkably increased the production of TGF-*β*1 in OVA-treated DCs, whereas the miR-223-3p inhibitor significantly reduced TGF-*β*1 production (both *P* < 0.01). However, miR-223-3p did not affect the secretion of IL-10, IL-12, and IL-6 (Figures 4(b)–4(d)).

**Figure 5:**
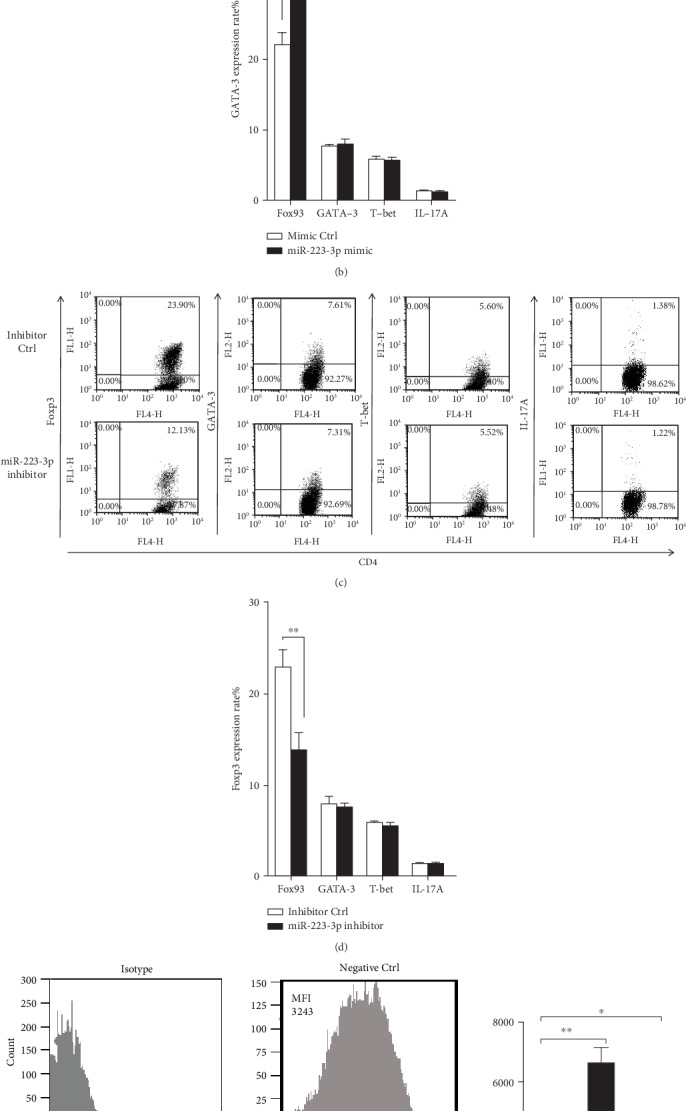
miR-223-3p regulated DC-initiated CD4+ T cell differentiation Immature mouse DCs were transfected with the miR-223-3p mimic (a, b), mimic control, miR-223-3p inhibitor (c, d), or inhibitor control, at a final concentration of 50 nM. After 24 h, DCs were stimulated with 100 *μ*g/ml OVA for 24 h. Purified CD4+ T cells were cocultured with the transfected DCs at a ratio of 1 : 10 (DC/T cells). After 4 days, cells were collected and stained with anti-CD4-APC, followed by intracellular staining with FITC-conjugated anti-Foxp3, FITC-conjugated anti-IL-17A, PE-conjugated anti-GATA-3, or PE-conjugated anti-T-bet, respectively. The cells were then analyzed by flow cytometry. The bar chart indicated the specific transcription factor of T cells in the gate of CD4+ cells in each group. Data were shown as mean ± SD of three independent experiments. *t*-test, ^∗^*P* < 0.05, ^∗∗^*P* < 0.01. (e) The effect of miR-223-3p on CD103 expression in OVA-treated DCs. Immature DCs were transfected with the miR-223-3p inhibitor (50 nM), miR-223-3p mimic (50 nM), or negative control (50 nM) for 24 h, respectively. DCs were incubated with OVA (100 *μ*g/ml) for 24 h, and cells were collected for flow cytometry analysis (gating for CD11c) to determine CD103 expression levels. (f) MFI value presented CD103 expression levels of the three groups. Data were presented as mean ± SD of three independent experiments. Post hoc Tukey's HSD test, ^∗^*P* < 0.05, ^∗∗^*P* < 0.01.

It is known that TGF-*β*1 plays an important role in Treg cell differentiation [19]. To investigate if miR-223-3p has an effect on the DC-mediated T cell differentiation, mixed lymphocyte reaction was performed. The miR-223-3p mimic significantly increased the ratio of Foxp3+ CD4+ T cells as compared with the control group (*P* < 0.01, Figure 5(a)) but did not affect the ratio of T-bet+, GATA-3+, and IL-17A+ CD4+ T cells (Figure 5(a)). Accordingly, miR-223-3p inhibition exhibited an opposite effect on the differentiation and polarization of T cells (*P* < 0.01, Figure 5(b)). These data suggested that miR-223-3p participated in the regulation of DC-mediated regulatory T cell polarization. The effect of miR-223-3p on CD103 (a tolerogenic marker) expression in OVA-treated DCs was investigated. As shown in Figures 5(e) and 5(f), the miR-223-3p mimic significantly upregulated CD103 in DCs, suggesting that miR-223-3p promoted tolerogenic DCs.

### 3.4. miR-223-3p Downregulated Rhob Protein Expression to Inhibit Antigen Endocytosis and Presentation of BMDCs, Followed by Treg Cell Differentiation

**Figure 6:**
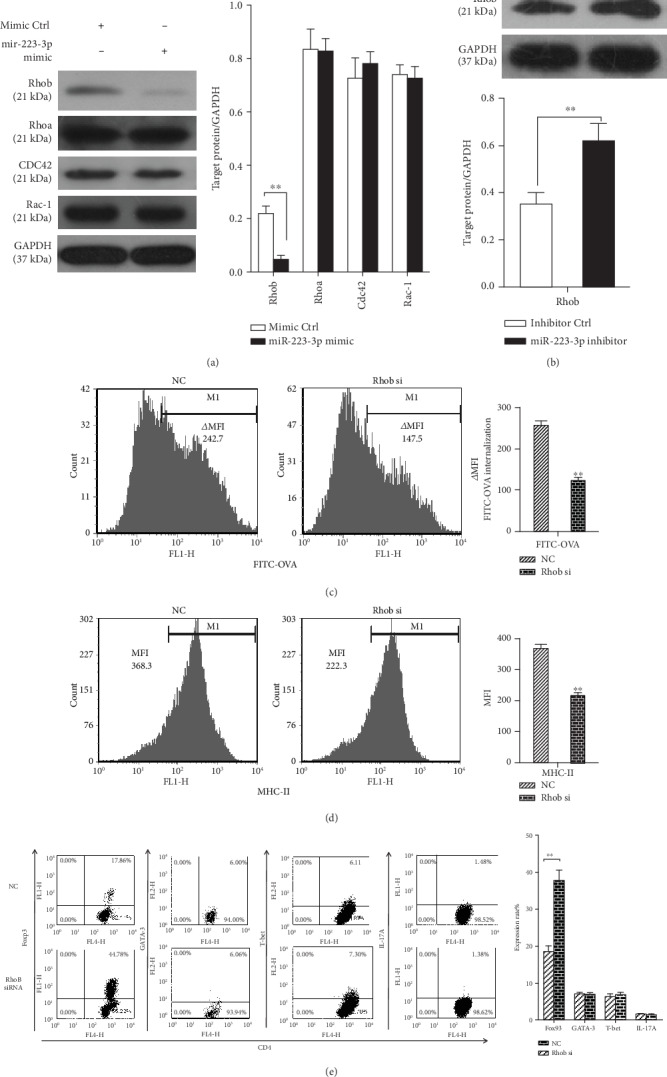
miR-223-3p suppresses Rhob expression to downregulate antigen endocytosis and presentation of BMDCs, followed by Treg cell polarization. (a, b) Mouse immature DCs were transfected with the miRNA mimic control, miR-223-3p mimic, miRNA inhibitor control, and miR-223-3p inhibition, at a final concentration of 50 nM. After 24 h, DCs were stimulated with 100 *μ*g/ml OVA for 24 h. Cell lysates were subjected to Western blot analysis for Rhob, CDC42, Rhoa, and Rac-1 proteins. (c–e) Immature mouse DCs were transfected with 50 nM siRNA-Rhob or siRNA control. (c) After 24 h of transfection, endocytic activity (FITC-OVA uptake) of BMDCs was measured by flow cytometry. (d) After 24 h of transfection, DCs were stimulated with 100 *μ*g/ml OVA for 24 h and then stained with MHC-II antibody, followed by flow cytometry analysis. The bar chart indicated the MFI in the gate of CD11c+ cells in each group. (e) After 24 h of transfection, DCs were stimulated with 100 *μ*g/ml OVA for 24 h. Purified CD4+ T cells were cocultured with the transfected DCs at a ratio of 1 : 10 (DC/T cells). After 4 d, cells were collected and stained with anti-CD4-APC, followed by intracellular staining with FITC-conjugated anti-Foxp3, FITC-conjugated anti-IL-17A, PE-conjugated anti-GATA-3, or PE-conjugated anti-T-bet, respectively. The cells were then analyzed by flow cytometry. The bar chart indicated the specific transcription factor of T cells in the gate of CD4+ cells in each group. Data were presented as mean ± SD of three independent experiments. *t*-test, ^∗^*P* < 0.05, ^∗∗^*P* < 0.01.

It has been shown that Rhob plays a key role in antigen endocytosis and presentation in BMDCs [23]. In addition, Rhob is a target of miR-223-3p [24]. Hence, we investigated if Rhob takes part in the antigen endocytosis and presentation in BMDCs. Western blot demonstrated that the miR-223-3p mimic decreased Rhob expression in OVA-induced DCs but did not affect the other members of the Rho family, including CDC42, Rhoa, and Rac-1 (Figure 6(a)). By contrast, the miR-223-3p inhibitor upregulated Rhob protein in OVA-treated DCs (Figure 6(b)).

Next, siRNA-Rhob was used for knockdown experiments. As shown in Supplementary Fig. S1B, siRNA-Rhob (50 nM) transfection effectively reduced the level of Rhob expression levels following OVA treatment. siRNA-Rhob knockdown significantly suppressed the ability of FITC-OVA endocytosis (*P* < 0.01, Figure 6(c)) and surface MHC-II molecule expression (*P* < 0.01, Figure 6(d)) in BMDCs. Rhob knockdown significantly promoted the preferential differentiation of Foxp3+ CD4+ T cells as compared with the control group (*P* < 0.01, Figure 6(e)) but did not affect T-bet+ cells, GATA-3+ cells, and IL-17A+ CD4+ T cells. These data suggested that miR-223-3p may target Rhob to regulate the antigen endocytosis and presentation in BMDCs and subsequent Treg responses.

### 3.5. Mannose Receptor-Mediated Antigen Endocytosis and Presentation Were Involved in miR-223-3p Modulation

**Figure 7:**
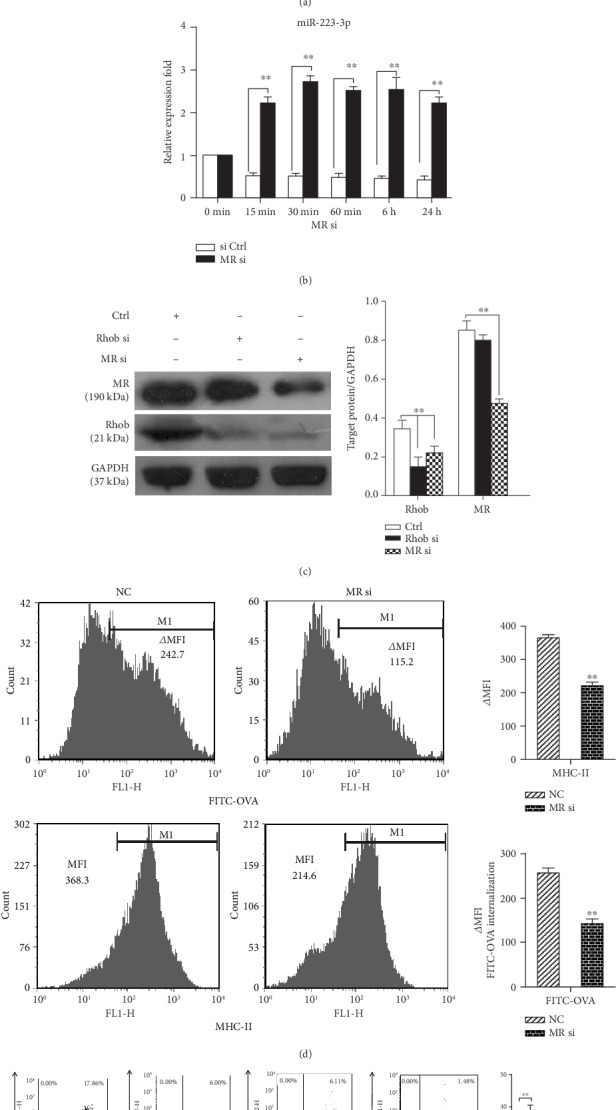
Mannose receptor-mediated antigen endocytosis and presentation were involved in miR-223-3p expression. (a) MR expression in DCs treated with OVA and miR-223-3p mimic or inhibitor was determined by Western blot. (b) Mouse immature DCs were transfected with siRNA-MR or siRNA control (50 nM) for 24 h, and the expression of miR-223-3p was detected by qRT-PCR. (c) Mouse immature DCs were transfected with siRNA-MR, siRNA-Rhob, or siRNA control (50 nM). After 24 h, DCs were stimulated with 100 *μ*g/ml OVA for 24 h. Cell lysates were subjected to Western blot for the mannose receptor, Rhob, and GAPDH. (d–f) Mouse immature DCs were transfected with siRNA-MR or siRNA control at a final concentration of 50 nM for 24 h. (d) DCs were incubated with FITC-OVA for 30 minutes, and the endocytic activity (FITC-OVA uptake) of BMDCs was measured by flow cytometry. For determining the MHC-II level, DCs were stimulated with 100 *μ*g/ml OVA for 24 h and then stained with MHC-II antibody, followed by flow cytometry analysis. (e) The transfected DCs were stimulated with 100 *μ*g/ml OVA for 24 h. Purified CD4+ T cells were cocultured with the transfected DCs at a ratio of 1 : 10 (DC/T cells). After 4 d, cells were collected and stained with anti-CD4-APC, followed by intracellular staining with FITC-conjugated anti-Foxp3, FITC-conjugated anti-IL-17A, PE-conjugated anti-GATA-3, or PE-conjugated anti-T-bet, respectively. The cells were then analyzed by flow cytometry. The bar chart indicated the specific transcription factor of T cells in the gate of CD4+ cells in each group. Data were presented as mean ± SD of three independent experiments. *t*-test, ^∗^*P* < 0.05, ^∗∗^*P* < 0.01.

The previous study demonstrated that human alveolar macrophages phagocytose Pneumocystis organisms predominantly mediated by CDC42 and Rhob activation and mannose receptors (MRs) [25]. Therefore, we investigated if MR is involved in the mechanism of miR-223-3p-regulated antigen endocytosis and presentation in DCs. To determine the effect of miR-223-3p on MR expression, the protein levels of MR in DCs treated with OVA and miR-223-3p mimic or inhibitor were determined by Western blot. As shown in Figure 7(a), the miR-223-3p mimic markedly inhibited MR expression, whereas the miR-223-3p inhibitor promoted MR expression in OVA-treated cells. Next, siRNA-MR was used for knockdown experiments. As shown in Supplementary Fig. S1B, siRNA-MR (50 nM) transfection effectively reduced endogenous MR expression levels. siRNA-MR knockdown significantly upregulated miR-223-3p from 15 min to 24 h (all *P* < 0.01, Figure 7(a)) and downregulated Rhob protein in OVA-treated DCs (Figure 7(b)). In addition, MR knockdown significantly inhibited the ability of FITC-OVA endocytosis (*P* < 0.01, Figure 7(c)) and surface MHC-II expression (*P* < 0.01, Figure 7(c)) as compared with the control group. Correspondingly, MR knockdown significantly promoted the preferential differentiation of Foxp3+ CD4+ T cells as compared with the control group but significantly decreased the ratio of T-bet+ cells, GATA-3+ cells, and IL-17A+ CD4+ T cells (Figure 7(e)).

## 4. Discussion

**Figure 8:**
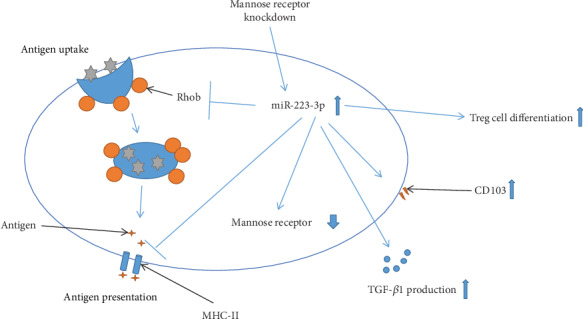
A schematic representation of the mechanism of miR-223-3p on regulating the antigen uptake and presentation capacities of BMDCs.

miR-223-3p has been shown to be involved in regulating the differentiation and function of DCs [18–20]. Bros et al. have demonstrated that mmu-miR-223-3p induces a protolerogenic state in BMDCs by attenuating the expression of its mRNA targets to control DC activation [18]. Ifergan et al. have reported that miR-223-3p regulates myeloid DC- (mDC-) induced activation of pathologic Th17 responses during autoimmune inflammation [20]. Zhou et al. have revealed that miR-223-3p regulates the differentiation and function of intestinal DCs and macrophages by targeting C/EBP*β* [19]. In this study, we investigated the effect of miR-223-3p on the antigen uptake and presentation capacities of BMDCs and the underlying molecular mechanism. The results showed that OVA treatment significantly downregulated miR-223-3p in BMDCs from 15 min to 24 h in a time-dependent manner. The miR-223-3p mimic significantly inhibited OVA-induced endocytosis and surface expression of MHC-II on BMDCs. miR-223-3p increased TGF-*β*1 production in OVA-treated DCs. Mixed lymphocyte reaction showed that the miR-223-3p mimic significantly increased the ratio of OVA-induced Foxp3+ CD4+ cells, suggesting the promotion of Treg cell differentiation. In addition, the miR-223-3p mimic significantly upregulated CD103 in DCs, indicating the promotion of tolerogenic DCs. The miR-223-3p mimic decreased Rhob expression in OVA-induced DCs. Rhob knockdown significantly suppressed OVA-induced endocytosis and MHC-II molecule expression in BMDCs, promoting the preferential differentiation of Foxp3+ CD4+ T cells. MR knockdown significantly upregulated miR-223-3p from 15 min to 24 h, downregulated Rhob protein in OVA-treated DCs, inhibited the OVA-induced endocytosis and MHC-II expression, and promoted the preferential differentiation of Foxp3+ CD4+ T cells. Taken together, these data suggested that miR-223-3p inhibited MR-mediated antigen uptake and presentation capacities of BMDCs and promoted Treg cell differentiation via targeting MR signaling and Rhob. A schematic representation of the mechanism of miR-223-3p on regulating the antigen uptake and presentation capacities of BMDCs is shown in Figure 8.

Our results found that OVA treatment downregulated miR-223-3p, and miR-223-3p inhibited OVA antigen uptake and presentation in BMDCs, suggesting that miR-223-3p plays an inhibitory role in the DCs. To the best of our knowledge, this is the first study reporting an inhibitory effect of miR-223-3p on the antigen endocytosis and presentation capacities of DCs. Our findings are in line with Naqvi et al.'s *in vitro* observations that miR-24, miR-30b, and miR-142-3p interfere with the uptake and processing of OVA antigen in DCs [15]. These results indicate an inhibitory function of these miRNAs in mediating antigen internalization and presentation. It is worth investigating if there is a correlation among the molecular mechanisms of the inhibitory function of these miRNAs within DCs.

It is well known that the antigen endocytosis and presentation in DCs are essential for the direct interaction of DCs with naive T lymphocytes and initialization of adaptive immune responses [26]. Meanwhile, DCs are also the key players in maintaining immune tolerance, which is mainly demonstrated by inducing Treg cell differentiation [27]. Since we found that miR-223-3p inhibited antigen endocytosis and presentation in DCs, we then investigated if miR-223-3p affects the tolerogenic potential of DCs. Notably, the mixed lymphocyte reaction showed that the miR-223-3p mimic significantly promoted Treg cell differentiation. Furthermore, our ELISA results showed that immature DCs secreted large amounts of TGF-*β*1, while the secretion was gradually decreased during DC maturation. However, miR-223-3p mimic treatment induced elevated TGF-*β*1 secretion and promoted the differentiation of Treg cells. The inhibition of miR-223-3p exhibited the opposite biological effect. It is known that DC-induced Treg cell differentiation is largely mediated by TGF-*β* [28]. Therefore, these results indicated that miR-223-3p mimic treatment may inhibit antigen endocytosis and antigen presentation but promote the immune tolerance function of DCs. On the other hand, our results also revealed that the miR-223-3p mimic upregulated CD103 in DCs. CD103 is a tolerogenic marker [29]. It is known that intestinal CD103+ DCs induce Treg differentiation and intestinal mucosa homing to control tolerance by production of retinoic acid and TGF-*β* [30]. Taken together, these findings suggested that miR-223-3p promoted the tolerogenic potential of DCs. However, the detailed molecular mechanism requires further investigation.

Rhob has been shown as a target of miR-223-3p [24] and is implicated in antigen presentation of BMDCs [23]. In this study, the miR-223-3p mimic markedly downregulated Rhob protein, confirming that Rhob is a target of miR-223-3p in DCs. Moreover, we found that Rhob-silenced BMDCs exhibited similar behaviors with those with the miR-223-3p mimic, including suppressed antigen-presenting ability, increased TGF-*β*1 production, and elevated Treg cell differentiation. These data implied that miR-223-3p may exert its modulating function in BMDCs by targeting Rhob. It has been shown that MR is crucial for antigen uptake and antigen presentation by DCs [31]. In the current study, we observed the miR-223-3p mimic downregulated MR in DCs, implying MR is a target of miR-223-3p. Nevertheless, we also found that MR knockdown significantly upregulated miR-223-3p. These data may indicate that MR is a target of miR-223-3p, but MR signaling may feedback inhibit miR-223-3p expression. However, the detailed molecular mechanism remains to be further investigated. Our results showed that MR knockdown also downregulated Rhob protein in BMDCs, suppressed antigen-presenting ability, and promoted Treg cell differentiation, indicating that miR-223-3p may inhibit MR-mediated antigen internalization and presentation capacities of BMDCs (Figure 8). However, more evidence is needed to support this notion.

There are still some limitations to this study. Although we found that miR-223-3p inhibited antigen-presenting function but promoted tolerogenic potential of DCs, the detailed molecular mechanism remains to be elucidated. In addition, these *in vitro* results should be further validated in an animal model. Although we found concordant results employing the miR-223-3p mimic, Rhob siRNA, and MR siRNA, the direct pathway has not been verified experimentally. For example, it remains to be determined if the miR-223-3p inhibitor reverses the effects of MR siRNA. All these limitations should be addressed in the following study.

## 5. Conclusions

In summary, our findings suggested that miR-223-3p has an inhibitory effect on the antigen uptake and presentation capacities of DCs and promotes Treg cell differentiation, which is, at least partially, through targeting MR signaling and Rhob. Our findings aid in understanding the modulating role of miR-223-3p in DC functions.

## Figures and Tables

**Table 1:** Sequences of the miR-223-3p mimic/inhibitor and siRNA.

Mimic/inhibitor	Sequence
miR-223-3p mimic	Sense: 5′-UGUCAGUUUGUCAAAUACCCCA-3′ Antisense: 5′-GGGUAUUUGACAAACUGACAUU-3′

miRNA mimic control	Sense: 5′-UUCUUCGAACGUGUCACGUTT-3′ Antisense: 5′-GGGUAUUUGACAAACUGACAUU-3′

miR-223-3p inhibitor	Sense: 5′-UGGGGUAUUUGACAAACUGACA-3′

miRNA inhibitor control	Antisense: 5′-CAGUACUUUUGUGUAGUACAA-3′

**Table 2:** Sequence of siRNAs.

siRNA	Sequence
siRNA-Rhob	Sense: 5′-GCUCAAGAGACUAUUGUUATT-3′ Antisense: 3′-UAACAAUAGUCUCUUGAGCTT-5′

siRNA-mannose receptor (MR)	Sense: 5′-GGUGGGUUAUUUACAAAGATT-3′ Antisense: 3′-UCUUUGUAAAUAACCCACCTT-5′

Negative control	Sense: 5′-UUCUUCGAACGUGUCACGUTT-3′ Antisense: 5′-GGGUAUUUGACAAACUGACAUU-3′

## Data Availability

The data used to support the findings of this study are included within the article.

## Supplementary Materials

Supplementary Materials Supplementary Fig. S1. Efficiency of the mimic and inhibitor. (A) Immature DCs were transfected with the miR-223-3p inhibitor (50 nM)/miRNA inhibitor control or miR-223-3p mimic (50 nM)/miRNA mimic control, respectively. After 24 h, DCs were incubated with OVA (100 *μ*g/ml) for 24 h, and miR-223-3p levels were determined by qRT-PCR. (B) Immature DCs were transfected (siRNA-Rhob (50 nM)/siRNA-MR (50 nM) or the corresponding negative control) for 24 h. DCs were then incubated with OVA (100 *μ*g/ml) for 24 h, and MRC1 and Rhob levels were determined by qRT-PCR. Data were presented as mean ± SD of three independent experiments. ^∗^*P* < 0.05, ^∗∗^*P* < 0.01. Ctrli: miRNA inhibitor control; mimic Ctrl: miRNA mimic control; Ctrl: negative control; Rhob si: Rhob siRNA; MR si: MR siRNA.
